# Brazilian strains of *Toxoplasma gondii* are controlled by azithromycin and modulate cytokine production in human placental explants

**DOI:** 10.1186/s12929-019-0503-3

**Published:** 2019-01-21

**Authors:** Priscila Silva Franco, Paula Suellen Guimarães Gois, Thádia Evelyn de Araújo, Rafaela José da Silva, Bellisa de Freitas Barbosa, Angelica de Oliveira Gomes, Francesca Ietta, Lara Affonso dos Santos, Maria Célia dos Santos, José Roberto Mineo, Eloisa Amália Vieira Ferro

**Affiliations:** 1 Laboratório de Imunofisiologia da Reprodução, Instituto de Ciências Biomédicas, Universidade Federal de Uberlândia, Av. Pará, 1720, Building: 2B, CEP, Uberlândia, 38405-320 Brazil; 2 Laboratório de Biologia Celular, Instituto de Ciências Biomédicas e Naturais, Universidade Federal do Triângulo Mineiro, Uberaba, Brazil; 3 Department of Life Sciences, University of Siena, Siena, Italy; 4 Laboratório de Imunoparasitologia, Instituto de Ciências Biomédicas, Universidade Federal de Uberlândia, Uberlândia, Brazil

**Keywords:** *Toxoplasma gondii*, Brazilian strains, Azithromycin, Villous explants

## Abstract

**Background:**

*Toxoplasma gondii* is a protozoan parasite that causes congenital toxoplasmosis by transplacental transmission. Parasite strains are genetically diverse and disease severity is related to the genotype. In Uberlândia city, Brazil, two virulent strains were isolated: TgChBrUD1 and TgChBrUD2. Congenital toxoplasmosis is more prevalent in South America compared to Europe, and more often associated with severe symptoms, usually as a result of infection with atypical strains.

**Methods:**

Considering that *T. gondii* has shown high genetic diversity in Brazil, the effectiveness of traditional treatment may not be the same, as more virulent strains of atypical genotypes may predominate. Thus, the aim of this study were to evaluate the Brazilian strain infection rate in human villous explants and the azithromycin efficacy with regard to the control of these strains compared to traditional therapy. Villi were infected with RH, ME49, TgChBrUD1 or TgChBrUD2 strains and treated with azithromycin, spiramycin or a combination of pyrimethamine plus sulfadiazine. The villous viability was analyzed by LDH assay and morphological analysis. Parasite proliferation, as well as production of cytokines was analyzed by qPCR and ELISA, respectively. Statistical analysis was performed using the GraphPad Prism 5.0.

**Results:**

The treatments were not toxic and TgChBrUD1 infected villi showed a higher parasite burden compared with others strains. Treatments significantly reduced the intracellular proliferation of *T. gondii*, regardless of the strain. TgChBrUD1-infected villi produced a larger amount of MIF, IL-6 and TGF-β1 compared with other infected villi. Azithromycin treatment increased MIF production by RH- or TgChBrUD2-infected villi, but in ME49- or TgChBrUD1-infected villi, the MIF production was not altered by treatment. On the other hand, azithromycin treatment induced lower IL-6 production by ME49- or TgChBrUD1-infected villi.

**Conclusions:**

Azithromycin treatment was effective against *T. gondii* Brazilian strains compared with conventional treatment. Also, the TgChBrUD1 strain replicated more in villi and modulated important cytokines involved in parasite control, showing that different strains use different strategies to evade the host immune response and ensure their survival.

## Background

Toxoplasmosis is a disease caused by *Toxoplasma gondii*, an obligate intracellular protozoan parasite that infects a wide range of hosts, including humans [1]. As an opportunistic human pathogen, *T. gondii* causes a devastating disease in immunocompromised individuals and congenitally infected neonates or children [2]. During pregnancy, the infection can result in the vertical transmission of *T. gondii* tachyzoites and the parasitic infestation can negatively affect the developing fetus [1]. The parasite reaches the fetus via the placenta, causing varying degrees of damage [3]. The frequency of congenital transmission increases according to the stage of the pregnancy, although the severity of the fetal infection decreases with this gestational progression [4, 5]. Most infected newborns have no symptoms at birth, but serious clinical manifestations can develop during childhood and early adulthood [6].

*T. gondii* was originally found as a clonal population derived in three lineages (I, II and III), predominantly observed in North America and Europe [7]. Genotypes not fitting within the three dominant lineages were classified as “atypical”, with a distinct genotype pattern having been demonstrated in Central and South America, where an abundance of different strain types has been found [8–11]. In Brazil, there are four clonal lineages, designated as BrI, BrII, BrIII, and BrIV [8, 12], and two parasite strains were obtained from chickens in Uberlândia city, Minas Gerais [13]. They were named the TgChBrUD1 strain, which exhibited the ToxoDB PCR-RFLP genotype #11 (also known as type BrII), and the TgChBrUD2 strain, which exhibited the ToxoDB PCR-RFLP genotype #6 (also known as type BrI and Africa 1).

The severity of the congenital disease is related to the gestational age at maternal infection, the concentration of parasites, the maternal/fetal immune response and the *T. gondii* type strain [14]. Recent research supports the concept that many atypical genotypes differ in pathogenicity and transmissibility from typical genotypes [14–16]. Because of the differential virulence of various *T. gondii* genotypes, the vertical transmission through the placenta and the immune responses might depend on strain variations [17]. Our previous studies have shown that these Brazilian strains (TgChBrUD1 and TgChBrUD2) are virulent in cells and animal models [13, 18, 19]. In addition, we demonstrated that re-infection with TgChBrUD1 or TgChBrUD2 atypical strains in rodents previously infected by the ME49 clonal strain promoted the vertical transmission of *T. gondii*, breaching the protection against congenital toxoplasmosis [19]. It means that atypical strains can present different behaviors in comparison to clonal strains, which shows the need for studies that clarify the immune mechanisms and treatment strategies against these strains.

In pregnant women with a confirmed diagnosis of toxoplasmosis through serology, PCR, utero ultrasound, or maternal clinical symptoms, anti-parasitic treatment is indicated [1]. Spiramycin is a macrolide antibiotic used to decrease the frequency of vertical transmission. However, if fetal infection occurs, a combination of pyrimethamine, sulfadiazine and folinic acid is used for the treatment of congenital toxoplasmosis [20, 21]. Pyrimethamine is a drug that prevents the growth and reproduction of *T. gondii* in cells. In the first trimester of gestation, this drug is not used, because of its potentially teratogenic properties toward the fetus. Thus, the folinic acid is used to prevent the hematological toxicities of pyrimethamine [1, 22, 23].

Azithromycin, another macrolide antibiotic, is a derivative of erythromycin with an anti-*Toxoplasma* effect [24–27]. It is widely used for the treatment of community-acquired pneumonia, *Chlamydia* during pregnancy, and has activity against *Plasmodium* spp. [28, 29]. Our previous studies have shown that azithromycin was able to control the vertical transmission of *T. gondii* in *Calomys callosus* rodents and in human BeWo trophoblast cells, demonstrating the important protective effect of azithromycin against this parasite in the maternal-fetal interface [24, 25]. In addition, in human villous explants from the third trimester of pregnancy infected with *T. gondii*, the treatment with azithromycin was able to control the replication of RH strain tachyzoites [27]. Another important effect of macrolide antibiotics is to exert anti-inflammatory activity and immunomodulatory effects [30–32]. Our previous study showed that azithromycin treatment induces an anti-inflammatory response in human BeWo trophoblastic cells infected by *T. gondii* [25].

Considering that *T.gondii* virulent strains are predominant in Brazil and the effectiveness of traditional therapy may not be the same, the present study aimed to evaluate the infection rate of these Brazilian strains in human villous explants from third trimester pregnancies, as well as the efficacy of azithromycin in the control of these strains compared to traditional therapy (spiramycin or the combination of sulfadiazine plus pyrimethamine).

## Methods

### Placenta samples and human villous explants culture

Third-trimester human placentas (36 to 40 weeks of pregnancy, *n* = 6) were collected after elective cesarean section deliveries at the Clinics Hospital of the Federal University of Uberlândia (HC-UFU), MG, Brazil. Exclusion criteria included pre-eclampsia, chronic hypertension, infectious disease including toxoplasmosis, chorioamnionitis, chronic renal disease, cardiac disease, connective tissue disease, pre-existing diabetes mellitus and gestational diabetes mellitus. Placental tissues were washed in ice-cold sterile PBS (pH 7.2) to remove excess blood, and then aseptically dissected using a stereomicroscope to remove endometrial tissue and fetal membranes up to 1 h after collection. Terminal chorionic villi containing five to seven free tips per explant were collected as described previously [27, 33, 34]. Explants were added to 96-well plates (one per well) and cultured in RPMI 1640 medium (Cultilab, Campinas, SP, Brazil) supplemented with 10% fetal bovine serum (FBS) (Cultilab), 100 U/mL penicillin, and 100 μg/mL streptomycin (Sigma-Aldrich Co., St. Louis, MO, USA) – complete medium at 37 °C and 5% CO_2_. The institutional ethics committee approved the study (Approval Number: 1.155.475).

### Parasite strains

Tachyzoites of RH, ME49, TgChBrUD1 or TgChBrUD2 strains were maintained in human trophoblast cells (BeWo line) cultured in RPMI medium containing 2% FBS, 100 U/mL penicillin, and 100 μg/mL streptomycin. The cell culture-derived parasites were stained with 0.4% Trypan blue and counted in a hemocytometric chamber to determine the concentrations of viable parasites to be used in experimental infection protocols.

### Antibiotics

Azithromycin (Biofarma, Uberlândia, MG, Brazil), spiramycin (Sigma) and a drug combination (PS) consisting of pyrimethamine (Sigma) and sulfadiazine (Sigma) were dissolved in DMSO (stock solution) to a concentration of 10,000 μg/mL for azithromycin, spiramycin, sulfadiazine and 3000 μg/mL for pyrimethamine. Stock solutions were freshly reconstituted and different drug concentrations were used for the treatment of villous explants.

### Tissue viability

In the first step of experiments, it was verified whether the drugs selected for treatment protocols could be toxic for placental explants. Tissue viability was analyzed by LDH assay and the tissue integrity by morphological analyses.

### LDH assay

In order to verify the toxicity of antibiotics in human villous explants, placental explants were evaluated for viability using an LDH assay [27].

The villous explants were collected and cultured in complete medium at 37 °C and 5% CO_2_. After 24 h, villi were treated with azithromycin (1000 μg/mL), spiramycin (1000 μg/mL) or a combination of sulfadazine plus pyrimethamine (200 μg/mL + 150 μg/mL, respectively). Drug concentrations were based on a previous study by our research group which showed parasite control with these doses [27]. In this study we treated the villous explants with different concentrations of azithromycin (200, 1000 or 5000 μg/mL) or PS (200, 1000 or 5000 μg/mL for pyrimethamine; 150, 750 or 3750 μg/mL for sulfadiazine). The concentrations of 200 or 1000 μg/mL did not cause significant cytotoxicity in villous explants and significantly reduced intracellular proliferation of *T. gondii* at both tested concentrations. Therefore, we choose the 1000 μg/mL concentration for experiments. As a control, villous explants were treated only with complete medium, corresponding to 100% viability. After 24 h of treatment, the supernatants were collected for the measurement of lactate dehydrogenase (LDH), according to the manufacturer’s instructions (LDH Liquiform, Labtest Diagnostica S.A., Lagoa Santa, MG, Brazil). Briefly, the supernatants collected were incubated with working reagent (NADH 360 μmol/L; sodium azide 0.095%; buffer 250 mmol/L; sodium pyruvate 6 mmol/L) for 1 min at 37 °C. Then, absorbance was measured in a DU-70 spectrophotometer (Beckman, Brea, CA., USA) at 340 nm, for 2 min. The enzyme LDH catalyzes the conversion of pyruvate to lactate in the presence of NADH. In addition, the decrease in absorbance at 340 nm due to the oxidation of NADH is proportional to the activity of LDH in the sample. Data were expressed as U/L of enzyme activity. Two placentas were used, and consequently, two independent experiments were performed in five replicates.

### Morphological analysis

In parallel, we evaluated the integrity of human villous explants after treatment with different antibiotics. For this purpose, villi were fixed in 10% buffered formalin, dehydrated in increasing alcohol concentrations, and finally embedded in paraffin. Then, 4 μm sections were made using a microtome and placed on glass slides. In the next step, villous sections were stained with hematoxylin and eosin, and morphological analyses were performed using a light microscope (BX40 Olympus, Tokyo, Japan) [27]. We evaluated morphological aspects of the syncytiotrophoblast and cytotrophoblast cells, as well as the mesenchyme. Two placentas were used and two independent experiments were performed in five replicates.

### Villous explants infection and treatments

Villous explants were collected and cultured in complete medium for 24 h. After, villi were infected with *T. gondii* tachyzoites (1 × 10^6^ parasites per well) of RH, ME49, TgChBrUD1 or TgChBrUD2 strains. After 24 h, the villous explants were washed with complete medium to remove non-adhered parasites and treated for an additional 24 h with azithromycin (AZ) (1000 μg/mL), spiramycin (ESP) (1000 μg/mL) or a combination of 200 μg/mL pyrimethamine plus 150 μg/mL sulfadiazine (PS) in complete medium. Infected/untreated villous explants were cultured with complete medium only and included as an experimental control. Afterwards, culture supernatants were collected and stored at − 80 °C for further cytokine detection. Villous explants were collected for morphological analysis or *T. gondii* intracellular proliferation assay by quantitative real-time PCR (qPCR). Four placentas were used, and consequently, four independent experiments were performed, at least, in duplicate.

### Quantitative real-time PCR

Total DNA was extracted from villous explants using the Wizard® Genomic DNA Purification Kit (Promega Co., Madison, WI, USA), according to the manufacturer’s instructions. Total DNA was quantified by UV spectrophotometry (ND1000 Spectrophotometer; NanoDrop Technologies, Wilmington, Delaware, USA). Real-time PCR was performed with a StepOnePlus® Real-Time PCR System (Applied Biosystems, Carlsbad, CA, USA) and QuantiNova SYBR Green PCR Kit (Qiagen), according to the manufacturer’s instructions. The reaction conditions followed the procedure described [35]. The primers (forward, 5′- TCCTCACCCTCGCCTTCAT-3′ and reverse, 5′-GCTCCTCCAGCCGTCTTG-3′) were engineered to detect the repetitive area of 529 bp in *T. gondii.* Positive and negative parasite controls were included in each assay. The reaction was carried out with 200 ng of DNA targets and a 100 ng DNA standard curve was concomitantly performed on each reaction in a seven time dilution series. The threshold cycle (Ct) value for each sample was compared to the standard control and the parasite quantity was analyzed. The data were presented in *T. gondii* DNA (100 ng/μl).

Furthermore, the mean ± SEM were used to express index of parasites replication, and the inhibition of growth parasites (%) in the presence of the drugs were calculated as follows: the average of parasite intracellular proliferation analyzed in untreated villous explants corresponded to 100%, then inhibition percentages (%) under drug treatments were calculated by subtracting the percentage values obtained in treated villous explants with each antibiotic from those obtained with untreated villous explants.

### Cytokines detection

The concentrations of cytokines were measured by sandwich ELISA. The IL-6, TNF-α, TGF-β1 (OpTEIA, BD Bioscience, San Diego, CA, USA) and MIF (Duoset R&D Systems, Minneapolis, MN, USA) cytokines were measured according to the manufacturer’s instructions. The concentrations of cytokines in culture supernatants were calculated from a standard curve of each human recombinant cytokine. The limit of detection was according to the last point of the standard curve (IL-6: 4.7 pg/mL; TNF-α: 7.8 pg/mL; TGF-β1: 125.0 pg/mL and MIF 7.8 pg/mL). The data regarding cytokines were normalized by the ratio between the concentration of cytokines in pg/mL and the concentration of total proteins from the Bradford assay in μg/mL, resulting in pg/μg of tissue.

### Statistical analysis

Statistical analysis was performed using the GraphPad Prism 5.0 (GraphPad Software Inc., San Diego, CA, USA). All data were expressed as mean ± standard error of the mean (SEM). The comparison between infected groups (Control) with infected and treated groups (AZ or ESP or PS) was analyzed by Student’s *t* test. The comparison of the data from treated or untreated strain groups were analyzed by the One-way ANOVA test and the Newman-Keuls post-test. Nonparametric data were analyzed by the Kruskal-Wallis test and Dunn’s Multiple Comparison post-test. Statistical significance was established when *P* < 0. 05.

## Results

### Villous explants maintain tissue viability after treatments

**Fig. 1:**
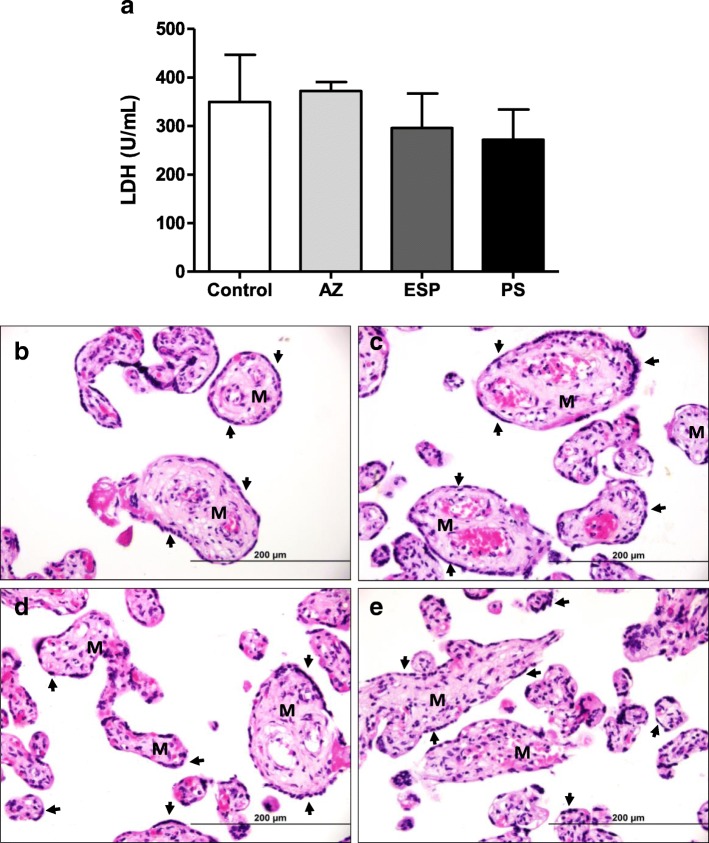
(**a**) Tissue viability in human villous explants. Villous explants supernatants were collected and lactate dehydrogenase (LDH) activity was measured using the LDH Liquiform kit. Villous explants were treated with azithromycin (1000 μg/mL) (AZ), spiramycin (1000 μg/mL) (ESP) or pyrimethamine (200 μg/mL) and sulfadiazine (150 μg/mL) (PS) for 24 h and the tissue toxicity was analyzed. Two placentas were used and two independent experiments were performed in five replicates. (ANOVA, *P* < 0.05). Representative photomicrographs of untreated human villous explants (**b**), or treated with azithromycin (**c**), spiramycin (**d**) or pyrimethamine and sulfadiazine (**e**). Arrows indicate syncytiotrophoblast layers and M indicates mesenchymal tissue. Haematoxylin and eosin stain. Bar scale: 200 μm

Villous explant viability after treatment with azithromycin (AZ), spiramycin (ESP) or pyrimethamine and sulfadiazine (PS) was evaluated using the LDH assay and morphological analysis (Fig. 1). No significant difference in LDH secretions was observed between uninfected/treated villous explants and uninfected/untreated villous explants (Fig. 1a). Also, treatment with either drug did not induce morphological alterations in villous explants (Fig. 1b-e). Syncytiotrophoblasts covering the chorionic villous were observed, but there were no morphological changes in the cytotrophoblasts and mesenchymal tissue (Fig. 1b-e).

### Villous explants infected with TgChBrUD1 strain present higher parasite burden and azithromycin reduces the tissue parasitism in all strain types

**Fig. 2:**
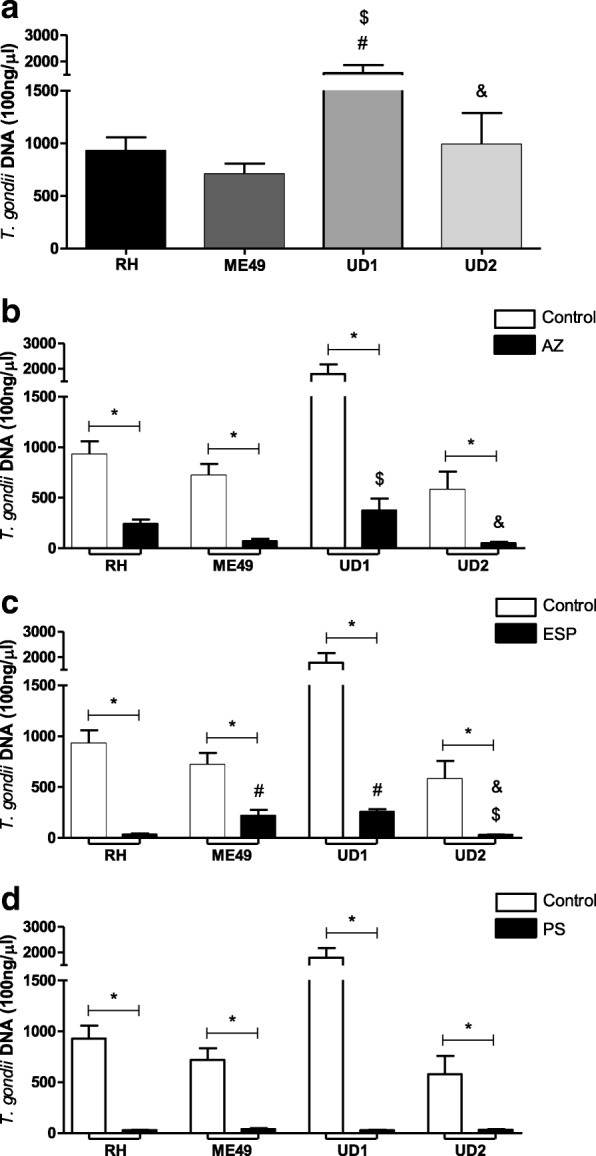
Parasite burden in infected human villous explants. The villous explants were infected with RH, ME49 or TgChBrUD1 (UD1) or TgChBrUD2 (UD2) (**a**) and treated with (**b**) azithromycin (1000 μg/mL) (AZ), (**c**) spiramycin (1000 μg/mL) (ESP) or (**d**) pyrimethamine (200 μg/mL) and sulfadiazine (150 μg/mL) (PS). The explants were collected and parasites were quantified at real-time PCR. The threshold cycle (Ct) value for each sample was compared to the standard control and the parasite quantity was analyzed. The data were presented in *T. gondii* DNA (100 ng/μl). Four placentas were used and four independent experiments were performed at least in duplicate. ^*^Comparison between untreated/infected villous explants and infected/treated villous explants (Student’s *t* test, mean ± standard error of the mean (SEM), *P* < 0.05); ^#^Comparison in relation to RH-infected villous explants; ^$^Comparison in relation to ME49-infected villous explants; ^&^Comparison in relation to UD1-infected villous explants; (ANOVA and Newman-Keuls multiple comparison test, mean ± standard error of the mean (SEM), *P* < 0.05)

The parasite intracellular proliferation in villous explants was determined using quantitative real-time PCR (Fig. 2). In the absence of treatment, TgChBrUD1-infected villous explants showed higher parasite burden when compared to RH-, ME49- or TgChBrUD2-infected villous explants (*P* < 0.05) (Fig. 2a). Treatment with either AZ or ESP or PS significantly reduced the intracellular proliferation of *T. gondii*, regardless *T. gondii* strain, in comparison to untreated villous explants (*P* < 0.05) (Fig. 2b-d). The villous explants infected with the TgChBrUD1 strain presented higher parasite burden compared to explants infected with the ME49 or TgChBrUD2 strain after AZ treatment (*P* < 0.05) (Fig. 2b). When treated with ESP, the villous explants infected with the ME49 or TgChBrUD1 strains presented higher parasite burden compared to explants infected with the RH or TgChBrUD2 strains (*P* < 0.05) (Fig. 2c). The treatment with PS was able to control all strains equally (*P* < 0.05) (Fig. 2d). The effect of drugs in parasite replication for each strain was also evaluated by *T. gondii* growth inhibition, as shown in Table 1. The treatments resulted in a significant inhibition of tachyzoite growth for all *T. gondii* strains. In addition, no significant difference was observed between drugs in infected villous explants, regardless of the strain (Table 1).

**Table 1:** Tachyzoite growth inhibition in infected villous explants after treatments

Strain/Drug	Parasite replication index^a^	Inhibition of growth parasites (%)^b^
RH/without treatment	927.9 ± 344.7	–
RH/AZ	241.1 ± 90.69^*^	74
RH/ESP	181.4 ± 168.7^*^	80
RH/PS	40,33 ± 25.58^*^	96
ME49/without treatment	705.4 ± 285.4	–
ME49/AZ	166.0 ± 164.7^*^	76
ME49/ESP	136,2 ± 110.7^*^	81
ME49/PS	35.75 ± 16.50^*^	95
UD1/without treatment	2095.0 ± 608.9	–
UD1/AZ	197.0 ± 232.1^*^	91
UD1/ESP	135.9 ± 135.6^*^	94
UD1/PS	21.45 ± 13.18^*^	99
UD2/without treatment	404.7 ± 226.4	–
UD2/AZ	42.50 ± 24.92^*^	89
UD2/ESP	26.53 ± 10.89^*^	93
UD2/PS	29.14 ± 16.13^*^	93

Tachyzoite growth inhibition in villous explants infected with RH, ME49, TgChBrUD1(UD1) or TgChBrUD2 (UD2) strains and treated with azithromycin (AZ), spiramycin (ESP) or pyrimethamine and sulfadiazine (150 μg/mL) (PS). ^a^Index of parasites replication was expressed in mean ± SD from three independent experiments in three replicates. ^b^Inhibition of growth parasites percentage in relation to untreated villous explants. ^*^Statistically significant differences in relation to the absence of treatment (control) *P* < 0.05

### MIF, IL-6 and TGF-β1 production is higher in TgChBrUD1-infected villous explants

**Fig. 3:**
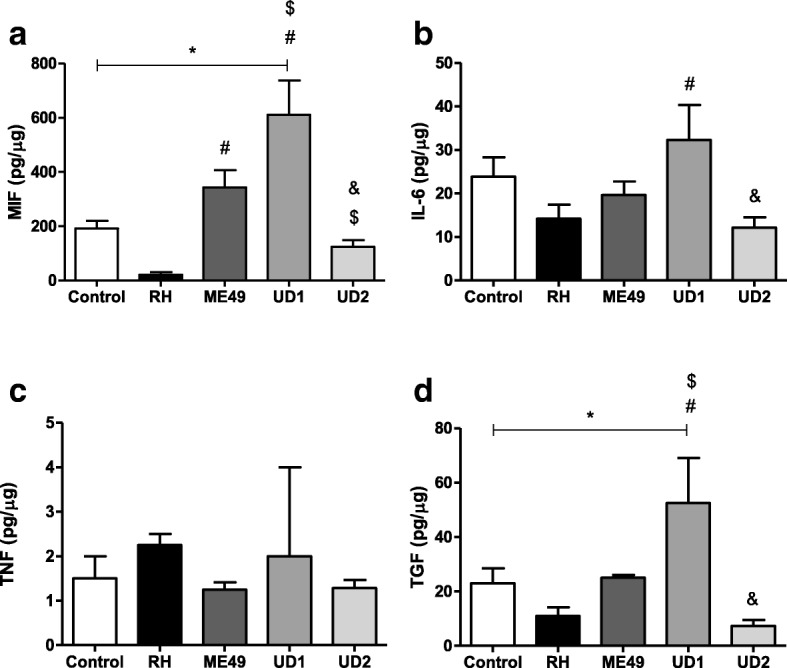
Cytokine production by human villous explants infected or not with RH, ME49, TgChBrUD1 (UD1) or TgChBrUD2 (UD2) strain. Supernatants were collected after 24 h of infection and the production of (**a**) MIF, (**b**) IL-6, (**c**) TNF and (**d**) TGF-β1 was measured by sandwich ELISA. Four placentas were used and four independent experiments were performed at least in duplicate. ^*^Comparison in relation to uninfected villous explants. ^#^Comparison in relation to RH-infected villous explants. ^$^Comparison in relation to ME49-infected villous explants. ^&^Comparison in relation to UD1-infected villous explants. MIF/IL-6/TGF-β (ANOVA and Newman-Keuls multiple comparison test, mean ± standard error of the mean (SEM), *P* < 0.05. TNF (Kruskal-Wallis and Dunn’s multiple comparison test, median with range, *P* < 0.05)

After evaluating the tissue parasitism in villous explants infected with different strains, we measured the cytokine levels (Fig. 3). Villous explants infected by RH, ME49 or TgChBrUD2 strains did not change the MIF, IL-6, TNF or TGF-β1 production in relation to uninfected villous (Fig. 3a-d). On the other hand, villous explants infected by TgChBrUD1 significantly increased MIF and TGF-β1 release in comparison to untreated villous (*P* < 0.05, Fig. 3a, d). In addition, TgChBrUD1 induced increased MIF, IL-6 and TGF-β1secretion in relation to villous explants infected by RH (*P* < 0.05, Fig. 3a, b and d). ME49 infection caused higher MIF production only when compared to RH-infected villi (*P* < 0.05, Fig. 3a). Furthermore, TgChBrUD1 triggered high MIF and TGF-β1 levels when compared to ME49-infected villi (*P* < 0.05, Fig. 3a, d). Villous explants infected with TgChBrUD2 induced lower MIF, IL-6 and TGF-β1 levels in comparison to TgChBrUD1 and, at the same time, triggered lower MIF production in relation to the ME49 strain (*P* < 0.05, Fig. 3a, b and d). Finally, no change in TNF production was observed for any experimental conditions (Fig. 3c).

### TgChBrUD1 and/or TgChBrUD2 strains modulate MIF production in treated villous explants

**Fig. 4:**
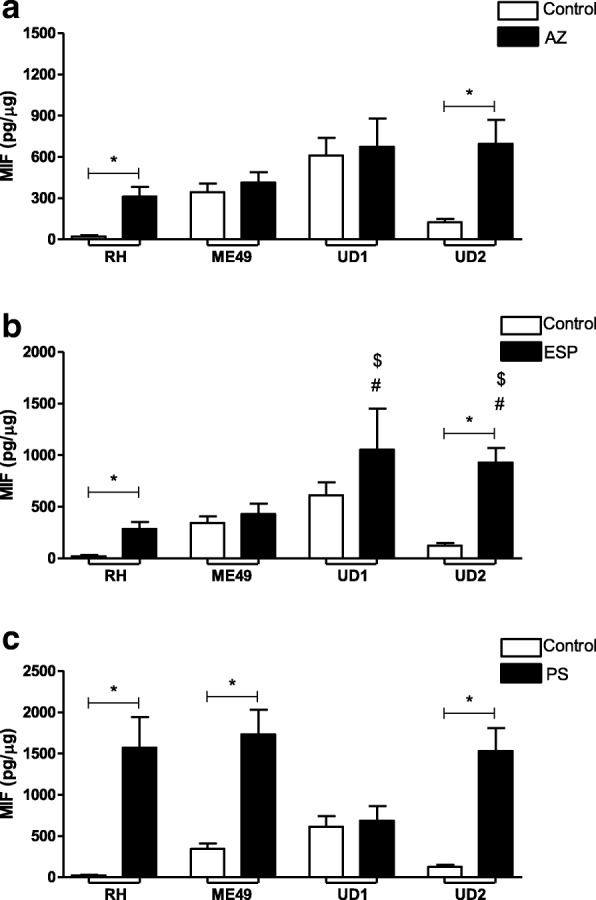
MIF production by human villous explants infected with RH, ME49, TgChBrUD1 (UD1) or TgChBrUD2 (UD2) strains and treated with (**a**) azithromycin (1000 μg/mL) (AZ), (**b**) spiramycin (ESP) (1000 μg/mL) or (**c**) pyrimethamine (200 μg/mL) and sulfadiazine (150 μg/mL) (PS). As a control, villous explants were only infected with RH, ME49, UD1 or UD2 (Control). Supernatants were collected after 24 h of infection and the production of MIF was measured by sandwich ELISA. Four placentas were used and four independent experiments were performed at least in duplicate. ^*^Comparison between infected villous explants and infected treated villous explants (Student’s *t* test, mean ± standard error of the mean (SEM), *P* < 0.05; ^#^Comparison in relation to RH-infected/treated villous explants; ^$^Comparison in relation to ME49-infected/treated villous explants; ^&^Comparison in relation to UD1-infected/treated villous explants (ANOVA and Newman-Keuls multiple comparison test, mean ± standard error of the mean (SEM), *P* < 0.05)

After verifying the cytokine profile in untreated villous explants infected with different *T. gondii* strains, we verified the cytokine release in infected villous explants treated with the panel of selected drugs (Fig. 4).

Villous explants treated with AZ and infected by either RH or TgChBrUD2 strains up-regulated MIF production in comparison to infected/untreated villi (*P* < 0.05, Fig. 4a). No significant differences were found between villous explants infected with ME49 or TgChBrUD1 strains and infected/untreated villi (Fig. 4a). Also, there were no significant differences in MIF production between infected villous explants, independent of the strain, after treatment with AZ (Fig. 4a).

Villous explants treated with ESP and infected by RH or TgChBrUD2 up-regulated the MIF production in comparison to infected/untreated villi (*P* < 0.05, Fig. 4b). No significant differences were found between villous explants infected with the ME49 or TgChBrUD1 strains and infected/untreated villi (Fig. 4b). ESP-treated villous explants infected with TgChBrUD1 or TgChBrUD2 increased the MIF release in relation to villi infected with ME49 or RH and treated with ESP (*P* < 0.05, Fig. 4b).

The PS treatment increased MIF production by villous explants infected with RH, ME49 or TgChBrUD2 in relation to untreated/infected villi (*P* < 0.05, Fig. 4c); however, there was no significant difference in MIF production by villous explants infected with TgChBrUD1 in relation to untreated/infected villi (Fig. 4c). There were no significant differences between infected villous explants, independent of the strain, after treatment with PS (Fig. 4c).

### IL-6 and TGF-β1 production were down-regulated by treatments in villous explants infected with ME49 or TgChBrUD1

**Fig. 5:**
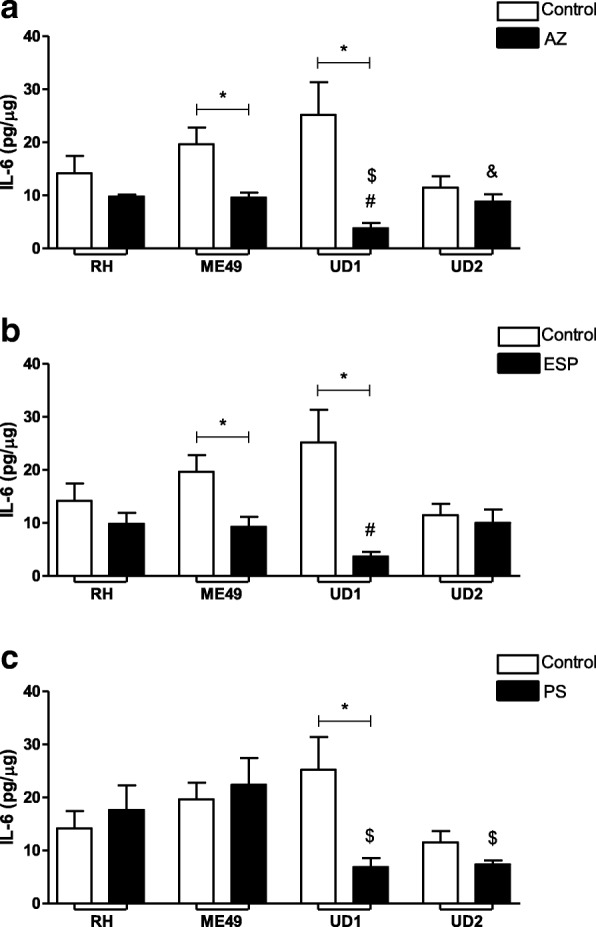
IL-6 production by human villous explants infected with RH, ME49 or TgChBrUD1 (UD1) or TgChBrUD2 (UD2) strain and treated with (**a**) azithromycin (1000 μg/mL) (AZ), (**b**) spiramycin (ESP) (1000 μg/mL) or (**c**) pyrimethamine (200 μg/mL) and sulfadiazine (150 μg/mL) (PS). As a control, villous explants were only infected with RH, ME49, UD1 or UD2 (Untreated). Supernatants were collected after 24 h of infection and the production of MIF was measured by sandwich ELISA. Four placentas were used and four independent experiments were performed at least in duplicate. ^*^Comparison between infected villous explants and infected treated villous explants (Student’s *t* test, mean ± standard error of the mean (SEM), *P* < 0.05; ^$^Comparison in relation to ME49-infected/treated villous explants; ^&^Comparison in relation to UD1-infected/treated villous explants (ANOVA and Newman-Keuls multiple comparison test, mean ± standard error of the mean (SEM), *P* < 0.05)

**Fig. 6:**
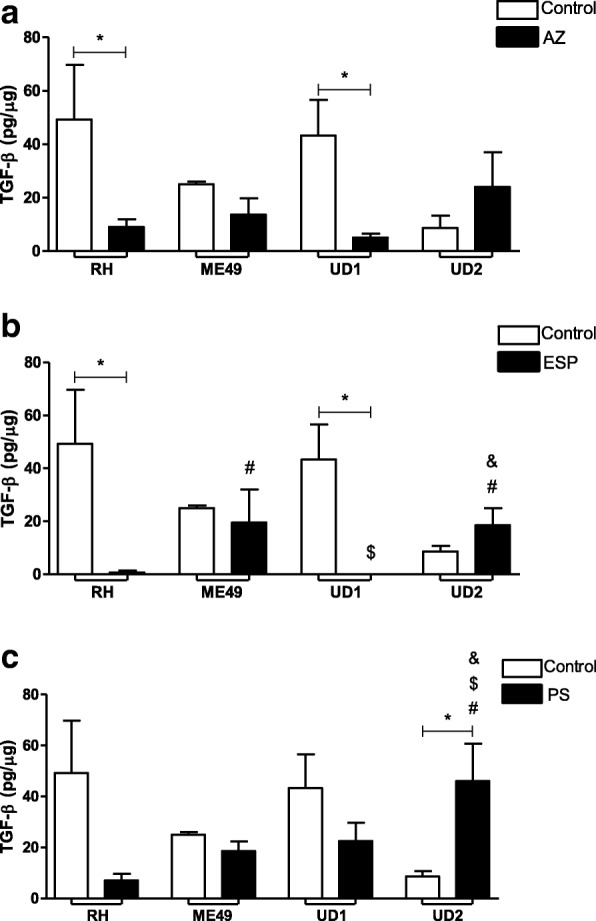
TGF-β production by human villous explants infected with RH, ME49 or TgChBrUD1 (UD1) or TgChBrUD2 (UD2) strain and treated with (**a**) azithromycin (1000 μg/mL) (AZ), (**b**) spiramycin (ESP) (1000 μg/mL) or (**c**) pyrimethamine (200 μg/mL) and sulfadiazine (150 μg/mL) (PS). As a control, villous explants were only infected with RH, ME49, UD1 or UD2 (Untreated). Supernatants were collected after 24 h of infection and the production of MIF was measured by sandwich ELISA. Four placentas were used and four independent experiments were performed at least in duplicate. ^*^Comparison between infected villous explants and infected treated villous explants (Student’s *t* test, mean ± standard error of the mean (SEM), *P* < 0.05; ^$^Comparison in relation to ME49-infected/treated villous explants; ^&^Comparison in relation to UD1-infected/treated villous explants (ANOVA and Newman-Keuls multiple comparison test, mean ± standard error of the mean (SEM), *P* < 0.05)

Also, the production of IL-6 and TGF-β1 was analyzed between infected villous explants (untreated group) and infected/treated villous explants (treated group) for each drug (Figs. 5 and 6).

The AZ and ESP treatments decreased the IL-6 production by villous explants infected with the ME49 or TgChBrUD1 strains in comparison to untreated/infected villi (*P* < 0.05, Fig. 5a-b). No significant differences were found between villous explants infected with RH or TgChBrUD2 strains and infected/untreated villi (Fig. 5a-b). The comparison between infected villous explants treated with AZ showed that TgChBrUD1 infected villous explants produced less IL-6 than RH, ME49 (*P* < 0.05) (Fig. 5a). In addition, villous treated with AZ and infected by TgChBrUD2 upregulated IL-6 release if compared to TgChBrUD1-infected and treated explants (*P* < 0.05) (Fig. 5a). When the infected villous explants were treated with spiramycin, lower IL-6 production by TgChBrUD1 infected villous explants was observed compared to RH-infected villous explants (*P* < 0.05) (Fig. 5b).

The PS treatment decreased IL-6 production by villous explants infected with the TgChBrUD1 strain (*P* < 0.05), but no significant differences were found in IL-6 production by villous explants infected with the RH, ME49 or TgChBrUD2 strains compared with infected villous explants (Fig. 5c). The TgChBrUD1 and TgChBrUD2 infected villous explants treated with PS produced less IL-6 than ME49-infected and treated villous explants (*P* < 0.05) (Fig. 5c).

When the TGF-β1 cytokine was analyzed, it was observed that treatment with AZ or ESP reduced TGF-β1 production by villous explants infected with the RH or TgChBrUD1 strain compared to infected and untreated villous explants (*P* < 0.05) (Fig. 6a and b).

In the AZ-treated group, no significant differences were observed in TGF-β production by infected villous explants, regardless of the strain (Fig. 6a). In the ESP-treated group, the ME49 and TgChBrUD2-infected villous explants produced higher levels of TGF-β1 than RH-infected villous explants (*P* < 0.05) (Fig. 6b). Furthermore, during ESP treatment, TgChBrUD1-infected villous explants produced lower TGF-β1 levels compared to ME49-infected villous explants, while TgChBrUD2-infected villous explants produced higher TGF-β1 levels compared with TgChBrUD1-infected villous explants (*P* < 0.05) (Fig. 6b). The treatment with PS increased TGF-β1 production in the TgChBrUD2-infected villous explants compared to the untreated group or the ME49-, RH- or TgChBrUD1-infected and treated villous explants (*P* < 0.05) (Fig. 6c).

## Discussion

Molecular studies of isolated strains of *T. gondii* from South America have shown a high frequency of atypical genotypes, suggesting a high level of diversity in *T. gondii* [36]. Researchers have suggested that many atypical genotypes differ in pathogenicity and transmissibility from typical genotypes [15, 16, 19, 37], since congenital toxoplasmosis is more prevalent in South America compared to Europe, and more often associated with severe symptoms, usually as a result of infection with atypical strains [38, 39]. Therefore, the effectiveness of treatment in North America and Europe may not be the same, as more virulent strains of atypical genotypes may predominate [40].

In the present study, a different infection rate was demonstrated for the Brazilian strain in third trimester human villous explants. TgChBrUD1-infected villous explants presented a higher parasite burden than RH-, ME49- or TgChBrUD2-infected villous explants and produced high MIF, IL-6 and TGF-β1 levels. Cells and animal models have already been used to determine the virulence of the Brazilian strain. In BeWo cells, it was observed that TgChBrUD2 infection induced lower infection index/replication compared to TgChBrUD1 [41]. In *C. callosus* rodents, it was observed that both males and females were highly susceptible to infection by the TgChBrUD1 or TgChBrUD2 strain, with mortality after 9 days of infection by the parasite [18]. In a previous study using the same animal model, it was shown that when groups of re-infected animals were compared, TgChBrUD2 re-infected females were more susceptible during pregnancy. This group presented lower survival, higher morbidity scores, an increased number of pregnant animals with fetal reabsorption and a high fetal loss rate [19]. Curiously, although the TgChBrUD1 and TgChBrUD2 strains belong to an atypical strain, they presented different parasite burdens. In addition, the cytokine production was also different in this study. TgCHBrUD1-infected villous explants produced high levels of MIF, IL-6 and TGF-β1, but TgCHBrUD2-infected villous explants produced low levels of these cytokines. This result suggests that, although the polymorphisms in the alleles of DNA fragments proposed by Su et al. [42] elucidate aspects of molecular epidemiology and population genetics, they underestimate the biological diversity between strains. In different experimental models, Brazilian strains had different behaviors.

Congenital toxoplasmosis can be reduced to a different degree when prenatal treatment is started in early pregnancy [43, 44]. The rapid initiation of therapy to the infected pregnant women is still important, because fetal infection follows shortly after maternal infection [43]. If fetal infection is confirmed by amniocentesis, or if the mother is infected in late pregnancy and there is a high risk of intrauterine infection, pyrimehtamine and sulfadiazine with folic acid should be used to reduce the risk of congenital toxoplasmosis [22, 45]. Thus, in the present study, the efficacy of AZ in the control of Brazilian strains compared to traditional therapy was investigated.

First, the drug concentrations used (1000 μg/mL for both AZ and ESP, and a combination of 200 μg/mL for pyrimethamine plus 150 μg/mL for sulfadiazine) were selected based on a previous study that showed the lowest toxicity in the placental villous for these doses [27]. As expected, villous explants maintained viability after treatments, with no morphological alterations observed.

In the present study, villous explants treated with either AZ, ESP or PS reduced the intracellular proliferation of *T. gondii*, independent of the strain, compared with untreated villous explants. Furthermore, AZ treatment inhibited the parasite in the same way as classical treatments, regardless of the strain. Previous studies by our research group demonstrated that AZ treatment controlled the tachyzoites replication in BeWo cells and villous explants from the third trimester of pregnancy infected with the RH strain [25, 27]. Macrolide antibiotics such as azithromycin bind to the 50S ribosomal subunit and prevent protein biosynthesis on the plastid ribosome, showing a characteristic “delayed death phenotype”, presumably due to their ability to induce a time-dependent reduction in the copy number of the apicoplast genome anti-parasitic activity [46, 47]. Thus, it was demonstrated that AZ controlled tachyzoites replication of the Brazilian strain in the same way as conventional drugs, showing an alternative drug for the prevention of toxoplasmosis by atypical strains. *T. gondii* elicits different innate immune responses and virulent strains fail to establish a life-long chronic infection, killing the host prematurely due to hyperinflammation or heavy parasite burden depending on the host [48–50]. In addition, because of the apparently different virulence of various *T. gondii* genotypes, studies showed altered immune responses against particular genotypes [51–54].

Thereby, this parasite seemingly has the ability to determine its own destiny by maximizing its persistence and minimizing host immunopathology [55]. In the present study, it was observed that villous explants infected with the TgChBrUD1 strain presented higher parasite burden than explants infected with other strains after AZ treatment. In the ESP treatment, the villous explants infected with ME49 or TgChBrUD1 presented a higher parasite burden compared to explants infected with the RH or TgChBrUD2 strains. The cytokine analysis showed high MIF levels produced by RH- or TgChBrUD2-infected villous explants, and no difference in the IL-6 level compared to the control after AZ or ESP treatment. On the other hand, no difference was observed in MIF production by villous explants infected with the ME49 or TgChBrUD1 strains and low IL-6 levels compared to the control after AZ or ESP treatment. MIF is a soluble pro-inflammatory cytokine released by activated immune-competent cells that may act as an activator of innate immunity, regulating subsequent adaptive immune responses and modulating some cells responses [56–58]. In a previous study, our group demonstrated that MIF production by the human first-trimester placenta is up-regulated by parasite antigens (STAg) and may play an essential role as an autocrine/paracrine mediator in placental infection by *T. gondii* [59]. Moreover, studies observed high MIF production in (BeWo) human trophoblast cells and human villous explants from first- and third-trimester pregnancies, demonstrating a potential control in the immune response to *T. gondii* infection at maternal-fetal interface [34, 60]. IL-6 is fundamental in the process of embryo implantation and a variety of cell populations are known to produce IL-6 in the placental microenvironment [61, 62]. This cytokine participates in the protective immune response against infectious agents and IL-6 has been demonstrated to be necessary for anti–*T. gondii* immunity [63], but an imbalance of IL-6 signaling through the gp130 receptor subunit could make IL-6 a pathological rather than a protective factor [64]. In the present study, the action of AZ or ESP associated with mechanisms of MIF can be hypothesized to explain the low parasite burden observed in villous explants infected with the RH or TgChBrUD2 strains. On the other hand, the low IL-6 level and the unchanged MIF production by villous explants infected with TgChBrUD1 after treatments can be associated with higher parasitism than other strains, but the action of drugs is able to control *T. gondii* infection. TGF-β1 seems to be important for dampening the inflammatory response and minimizing the damage caused by inflammation in TgChBrUD1-infected villous explants, but no relationship with treatments was observed in the present study. It is important to emphasize that macrolide antibiotics have anti-inflammatory activity and immunomodulatory effects [32] that may have influenced cytokine production by villous explants.

Interestingly, the TgChBrUD2 (genotype 6 type BrI Africa 1) strain presented nine type I alleles out of 12 genetic markers and killed infected animals within a short period of time. In contrast, the TgCHBrUD1 (genotype 11 type BrII) strain showed five markers with the type I allele, two type II alleles and five markers of type III alleles [13]. In the present study, the analysis of parasitism and cytokine production by villous explants infected by clonal or Brazilian strains showed similar results to the RH and TgChBrUD2 strains. The same phenomenon was observed for ME49 and TgChBrUD1. It has been suggested that distinct combinations of alleles in several loci may be responsible for the heterogeneity observed in the virulence phenotype of *T. gondii* strains [65].

Studies by our research group demonstrated the relationship between apoptosis modulation during *T. gondii* infection and the virulence characteristics of the parasite. It was observed that the incidence of apoptosis in BeWo cells was differentially modulated by highly (RH) or moderately virulent (ME49) strains of *T. gondii,* since RH-infected BeWo cells had a lower incidence of apoptosis compared to the ME49 strain [66]. Therefore, ME49-infected BeWo cells exhibited a predominantly pro-inflammatory response profile, with the higher secretion of MIF, TNF-α, IL-12, IL- 17A and IL-6, whereas RH-infected cells showed a higher production of anti-inflammatory cytokines such as TGF-β and IL-10 [67]. Thus, the highly (RH and TgChBrUD2) or moderately (ME49 and TgChBrUD1) virulent strains can modulate, differently, important defense mechanisms in hosts to sustained intracellular survival. In the present study, it is possible to associate the cytokine production and parasite burden with the virulence of strains, as virulent strains induced less MIF and TGF-β production than strains with moderate virulence. Therefore, the treatments also influenced the cytokine production and parasitism, since differences in cytokine production were observed after treatments and there was a lower parasite burden than in untreated villous explants.

## Conclusion

In conclusion, the azithromycin treatment was as effective as the conventional treatment of human placental villi infected with *T. gondii*, regardless of the strain, suggesting that it may be an alternative drug for the prevention of congenital infection. In addition, the TgChBrUD1 strain was able to replicate more in villous explants than other strains and modulate important cytokines involved in parasite control, showing that different strains have different strategies to evade the host immune response and ensure survival.

## Abbreviations

AZ: Azithromycin
DMSO: Dimethyl sulfoxide
ELISA: Enzyme-Linked Immunosorbent Assay
ESP: Spiramycin
FBS: Fetal bovine serum
HC-UFU: Clinics Hospital of the Federal University of Uberlândia
IL-6: Interleukin 6
LDH: Lactate Dehydrogenase
MIF: Macrophage migration inhibitory factor
PS: pyrimethamine and sulfadiazine
qPCR: quantitative real-time PCR
STAg: Soluble tachyzoite antigen
TGF-β1: Transforming growth factor beta
TNF-α: Tumor necrosis factor
